# Application of Antibodies to Neuronally Expressed Nogo-A Increases Neuronal Survival and Neurite Outgrowth

**DOI:** 10.3390/ijms21155417

**Published:** 2020-07-30

**Authors:** Vini Nagaraj, Thomas Theis, Anmol Singh Johal, Arihant Seth, Jada Gore, Neha Arsha, Mukti Patel, Helen Baixia Hao, Nikki Kurian, Melitta Schachner

**Affiliations:** Keck Center for Collaborative Neuroscience and Department of Cell Biology and Neuroscience, Rutgers University, Piscataway, NJ 08554, USA; vn149@dls.rutgers.edu (V.N.); theis@dls.rutgers.edu (T.T.); anmolj555@gmail.com (A.S.J.); as2200@scarletmail.rutgers.edu (A.S.); gorejada100@gmail.com (J.G.); nehaarsha7@gmail.com (N.A.); myp29@scarletmail.rutgers.edu (M.P.); helenhao.bio@gmail.com (H.B.H.); nikkikurian@gmail.com (N.K.)

**Keywords:** Nogo-A, antibody, neurite outgrowth, cell culture, mouse, signal transduction, stress

## Abstract

Nogo-A, a glycoprotein expressed in oligodendrocytes and central nervous system myelin, inhibits regeneration after injury. Antibodies against Nogo-A neutralize this inhibitory activity, improve locomotor recovery in spinal cord-injured adult mammals, and promote regrowth/sprouting/saving of damaged axons beyond the lesion site. Nogo-A is also expressed by neurons. Complete ablation of Nogo-A in all cell types expressing it has been found to lead to recovery in some studies but not in others. Neuronal ablation of Nogo-A reduces axonal regrowth after injury. In view of these findings, we hypothesized that, in addition to neutralizing Nogo-A in oligodendrocytes and myelin, Nogo-A antibodies may act directly on neuronal Nogo-A to trigger neurite outgrowth and neuronal survival. Here, we show that polyclonal and monoclonal antibodies against Nogo-A enhance neurite growth and survival of cultured cerebellar granule neurons and increase expression of the neurite outgrowth-promoting L1 cell adhesion molecule and polysialic acid. Application of inhibitors of signal transducing molecules, such as c-src, c-fyn, protein kinase A, and casein kinase II reduce antibody-triggered neurite outgrowth. These observations indicate that the recovery-promoting functions of antibodies against Nogo-A may not only be due to neutralizing Nogo-A in oligodendrocytes and myelin, but also to their interactions with Nogo-A on neurons.

## 1. Introduction

Myelin-associated inhibitory molecules expressed in the adult central nervous system of mammals prevent axonal regrowth/sprouting and reconstruction of networks. Inhibitory molecules reduce restoration of disturbed networks after injury [1]. Among these inhibitory molecules, Nogo-A was the first to be identified as an important player. Nogo-A, the largest isoform in the *Nogo* gene family, which also includes Nogo-B and Nogo-C, is a guidance molecule that was initially described as a potent inhibitor of neuritogenesis and regeneration after central nervous system injury in adult mammals [2,3].

Many studies [4,5,6,7] have shown the growth cone-repellant activity of Nogo-A expressed by oligodendrocytes and myelin in vivo using Nogo-A knock-out and knock-in mice and in vitro using cultured mutant cells [8,9]. However, other studies [10] did not report these growth cone-repelling effects with similar transgenic animals with or without Nogo-A expression. The reason for this discrepancy is not clear. 

Certain types of neurons express Nogo-A, where it may regulate neurite outgrowth and fasciculation of axons during development [2,11,12]. Neuronal Nogo-A can reduce dendritic branching and synaptic functions, as indicated by studying complete Nogo-A knockout mice that do not express this molecule in oligodendrocytes or neurons throughout development [5]. 

Retinal ganglion cells have reduced capacity to regrow and sprout after optic nerve trauma in conditional knockout mice that do not express Nogo-A in neurons, with this effect being attributed to a *cis*-interaction of Nogo-A with its receptor at the cell surface to counteract negative effects of Nogo-A *trans*-interaction with glial cells [6]. To further investigate these findings, we applied polyclonal and monoclonal antibodies against Nogo-A to a highly enriched neuronal cell culture to see if the antibodies would enhance neurite outgrowth and neuronal survival. In these experiments, glia that may express Nogo-A are a very small minority and thus do not interfere with the neuronal Nogo-A effects. Cerebellar granule cells are widely chosen as a neuronal prototype, which comprises all the features necessary for probing the functions of a particular molecule. These cells can readily be obtained from early postnatal mice in sufficient amounts for biochemical experiments [13]. This prototype culture system has been successfully used in many studies and has served to discover many important cellular and molecular aspects of nervous system functions in vitro. In the context of Nogo-A research, these cells have been used to study Nogo-A functions [12].

In the present study, we show that monoclonal and polyclonal antibodies against Nogo-A enhanced survival of stressed neurons and promoted neuritogenesis. Inhibitors of signal transduction molecules known to affect neurite growth reduced antibody-induced neuronal survival and neuritogenesis. Nogo-A antibodies increased survival of stressed neurons and enhanced expression of the regeneration-conducive adhesion molecule L1 and polysialic acid. These observations suggest that Nogo-A antibodies applied in vivo not only neutralize the inhibitory functions of Nogo-A expressed in oligodendrocytes and myelin, but also directly and positively enhance regeneration of neurons.

## 2. Results

### 2.1. Nogo-A Is Expressed in Cultured Cerebellar Granule Neurons

**Figure 1:**
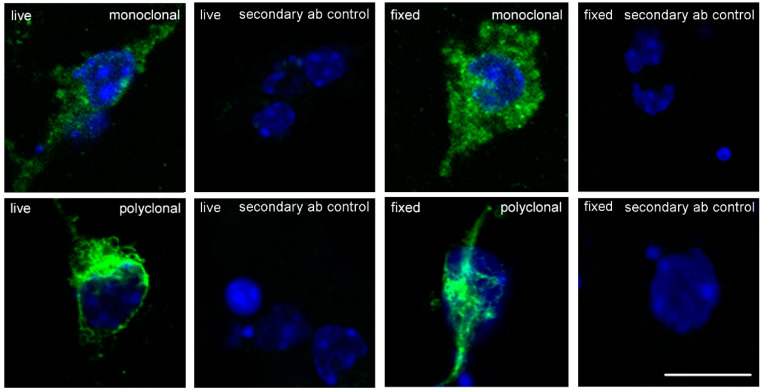
Nogo-A immunostaining of cultured cerebellar granule neurons. Representative images show cells immunostained for Nogo-A (green) and cell nuclei (blue). At 24 h after seeding, cells were either fixed before addition of Nogo-A antibodies, or live cells were incubated first with Nogo-A antibodies, and then incubated with the corresponding secondary antibodies coupled with Alexa Fluor 488. For control, secondary antibodies were applied without primary antibody. Thereafter, cells were stained with DAPI. Scale bar represents 10 μm for all images.

To determine the expression of Nogo-A, cerebellar granule cells were either fixed before application of Nogo-A antibodies or incubated live with Nogo-A antibodies and subsequently fixed. Monoclonal and polyclonal antibodies showed cell surface expression of Nogo-A on live and also on fixed cultured cerebellar granule neurons (Figure 1). Polyclonal antibodies yielded more intense staining than monoclonal antibodies at a similar concentration, since they can be assumed to recognize more epitopes than the monoclonal antibody and since secondary antibodies may have different potencies. Cells that were incubated only with the corresponding secondary antibodies did not show any immunofluorescence reactivity (Figure 1).

### 2.2. Nogo-A Antibodies Are Not Neurotoxic

**Figure 2:**
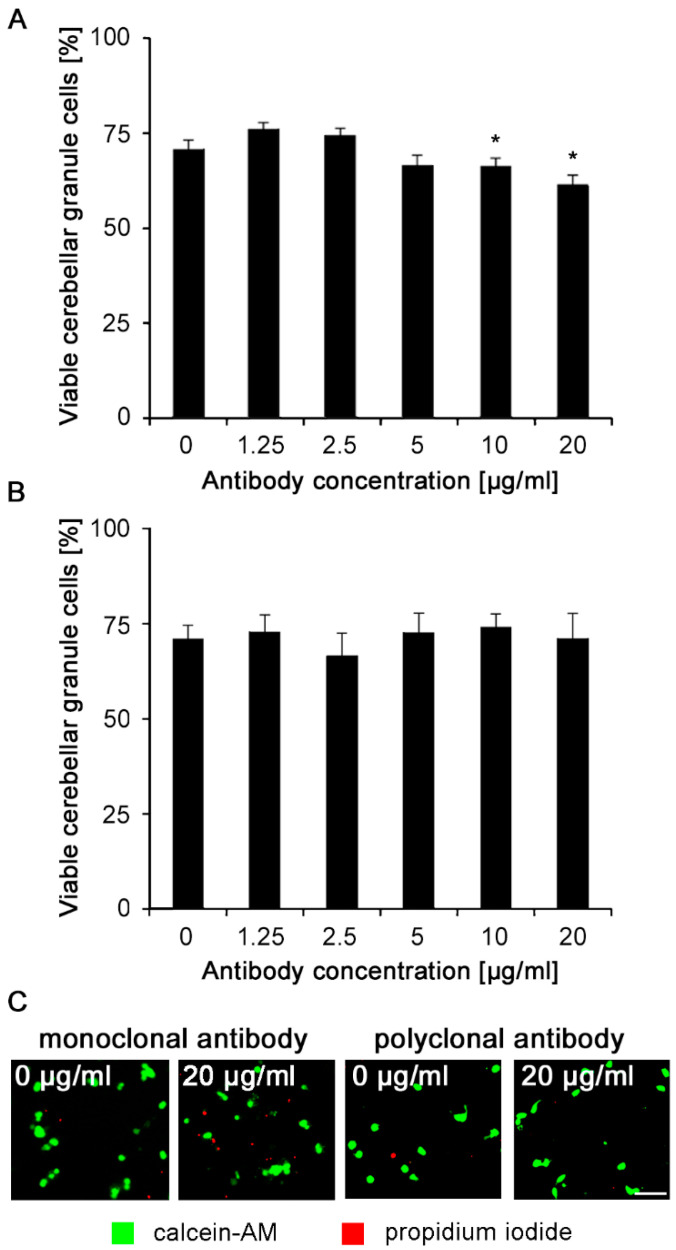
Monoclonal and polyclonal Nogo-A antibodies do not influence neuronal survival of cerebellar granule neurons maintained under physiological culture conditions. Cultures were treated with different concentrations of (**A**) monoclonal or (**B**) polyclonal Nogo-A antibodies for 24 h. Cell survival was then measured by propidium iodide (red)/calcein staining (green) (12 wells were analyzed for each treatment in three independent experiments). (**C**) Representative images of cells treated with 0 and 20 μg/mL antibodies. We used a macro in ImageJ to count cells. If the fluorescence intensity of one cell is not uniform, the program may detect two or more cells instead of one cell. Thus, we had to overexpose the image to the point where fluorescence intensity in the cell body is uniform and the cell body is distinct from its surrounding, less intensely labeled, and less organized structures. Data represent mean + SEM. * p < 0.05 difference to untreated control (0 μg/mL), one-way ANOVA with Tukey’s post-hoc test.

To exclude toxic effects and to determine the optimal concentrations for further experiments, cells were incubated for 24 h at concentrations between 1.25 and 20 μg/mL of monoclonal or polyclonal Nogo-A antibodies. At 10 and 20 μg/mL concentrations, cell viability was slightly lower, when compared to the vehicle-treated control, possibly due to traces of sodium azide (Figure 2A). Polyclonal Nogo-A antibody did not reduce cell viability at any of the concentrations tested (1.25 to 20 μg/mL) (Figure 2B). We conclude that under physiological culture conditions neither polyclonal nor monoclonal antibodies are toxic and thus do not affect cell survival at the lower concentrations used to analyze neurite outgrowth. 

### 2.3. Nogo-A Antibodies Stimulate Neurite Outgrowth 

**Figure 3:**
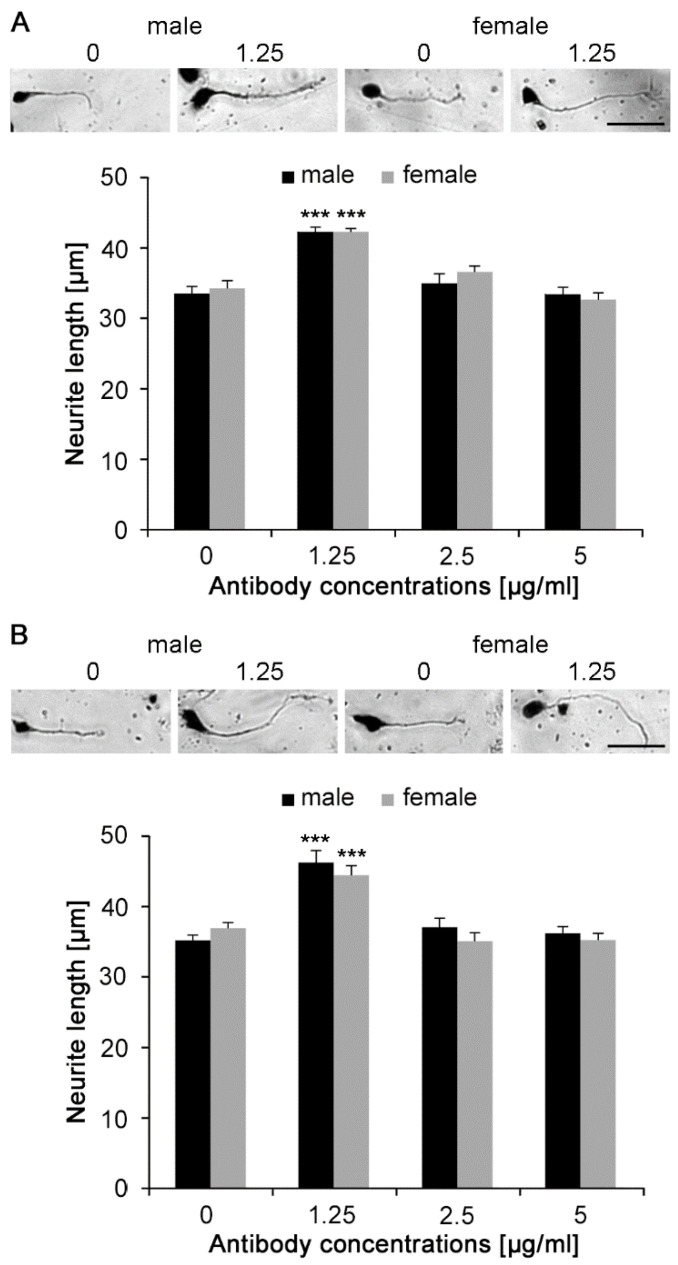
Monoclonal and polyclonal Nogo-A antibodies stimulate neurite outgrowth of cultured cerebellar granule neurons maintained under physiological conditions. Cultures were treated with different concentrations of (**A**) monoclonal or (**B**) polyclonal Nogo-A antibodies for 24 h. Total neurite length per cell was then determined (300 neurons were analyzed for each treatment in three independent experiments). Representative images are shown in the upper panels of (**A**) and (**B**). Data represent mean  +  SEM. *** *p* < 0.001 difference to untreated control, one-way ANOVA with Tukey’s post-hoc test. Scale bars represent 30 μm for all images.

Antibodies were then tested in neurite outgrowth experiments at concentrations of 1.25, 2.5, and 5 μg/mL (Figure 3). Neurite outgrowth was measured 24 h after addition of monoclonal and polyclonal antibodies. After longer culture times, neurites would be too long and thereby touch neighboring structures, preventing the accurate measurement of neurite lengths. Occasional neurites that had touched neighboring cells at 24 h were excluded from the analysis. Application of monoclonal antibody (Figure 3A) to the cultures increased neurite lengths from approximately 33 μm per neurite without antibody to approximately 43 μm in the presence of 1.25 μg/mL monoclonal antibody (*p* < 0.001) in both male and female cells. At 2.5 and 5 μg/mL, neurite lengths were similar to those without antibody, a phenomenon often observed when overstimulation occurs, even when in this case cell viability was not affected at this concentration (Figure 2). Polyclonal antibody (Figure 3B) also stimulated neurite outgrowth at 1.25 μg/mL in both male and female cells (*p* < 0.001). 

### 2.4. Nogo-A Antibodies Promote Cell Survival under Oxidative Stress Conditions

**Figure 4:**
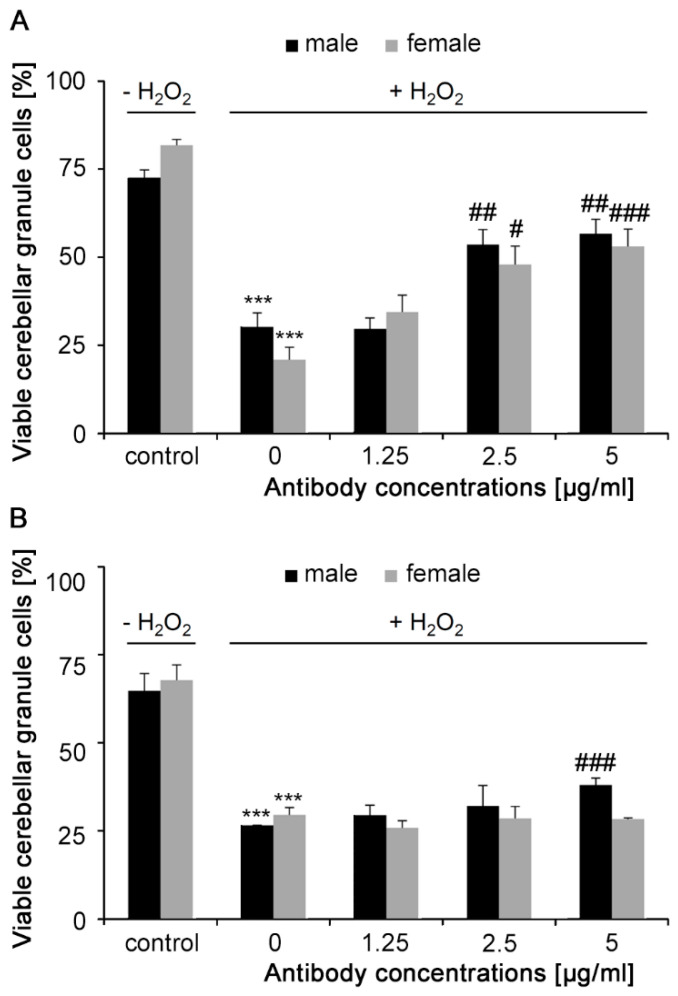
Monoclonal and polyclonal Nogo-A antibodies stimulate survival of stressed cultured cerebellar granule neurons. Cultures were treated with different concentrations of (**A**) monoclonal or (**B**) polyclonal Nogo-A antibodies for 3 h. Cell death was induced by addition of 10 μM H_2_O_2_ to the culture medium for 24 h. Cell survival was measured by propidium iodide-calcein staining (12 wells were analyzed for each treatment in three independent experiments). Data represent mean  +  SEM. *** *p* < 0.0001 difference to medium control and # *p* <  0.01, ## *p* < 0.001, ### *p* < 0.001 difference to control not treated with antibody, one-way ANOVA with Tukey’s post-hoc test.

Cerebellar granule neurons were stressed by application of H_2_O_2_ for 24 h, which leads to a significant reduction in cell survival of male and female cells (Figure 4). Incubation of cells for 3 h with monoclonal antibody increased cell survival at 2.5 and 5 μg/mL (Figure 4A) in male (53.6% and 56.7%, respectively) and female (47.9% and 53.1%, respectively) in comparison to cells not incubated with antibodies (male: 29.7%; female: 34.4%). Polyclonal antibody increased cell survival only for male cells (39.7%), but not for female (29.7%) cells, and only at a concentration of 5 μg/mL, compared to H_2_O_2_-treated controls (male: 27.8%; female: 30.89%) (Figure 4B). These results show that monoclonal and polyclonal Nogo-A antibodies reduce stress toxicity. 

### 2.5. Polyclonal Nogo-A Antibody Increases the Expression of L1 Protein and Polysialic Acid Glycan

**Figure 5:**
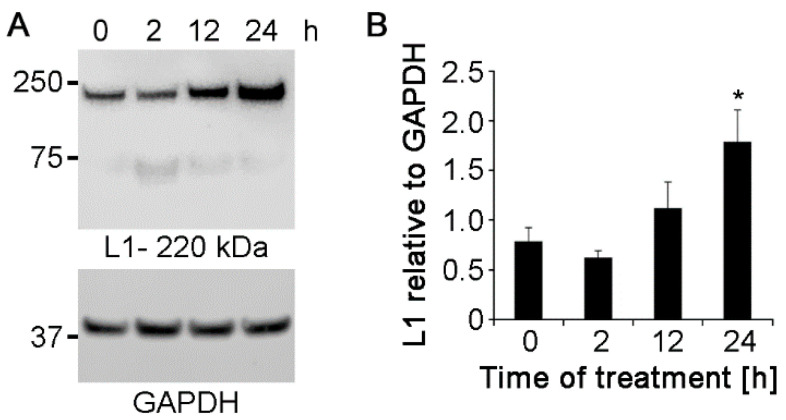
Polyclonal Nogo-A antibody increases L1 levels in cultures of cerebellar granule neurons. (**A**) Cultures maintained under physiological conditions were treated with 5 μg/mL polyclonal Nogo-A antibody and evaluated for L1 expression after 0, 2, 12, and 24 h by western blot analysis using antibody 172-R (seven cultures were analyzed for each treatment in seven independent experiments). (**B**) GAPDH was used for normalization. Data represent mean  +  SEM. * *p* < 0.001 difference to 0 h control, one-way ANOVA with Tukey’s post-hoc test.

**Figure 6:**
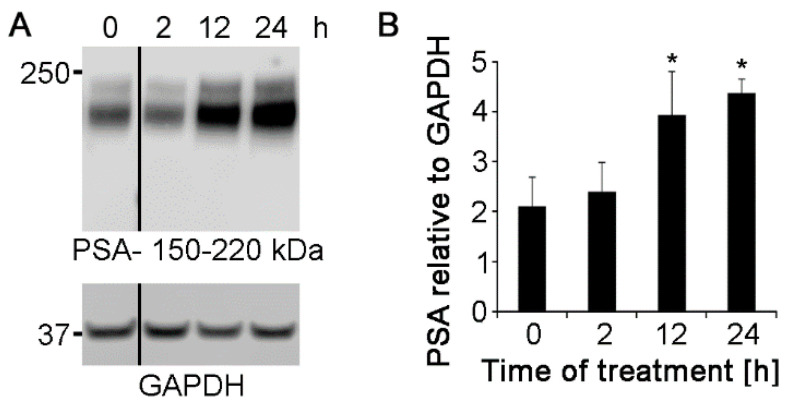
Polyclonal Nogo-A antibody increases polysialic acid (PSA) levels in cultures of cerebellar granule neurons. (**A**) Cultures maintained under physiological conditions were treated with 5 μg/mL polyclonal Nogo-A antibody and PSA expression was evaluated after 0, 2, 12, and 24 h by western blot analysis using PSA antibody 735 (seven cultures were analyzed for each treatment in seven independent experiments). (**B**) GAPDH was used for normalization. Data represent mean  +  SEM. * *p* < 0.05 difference to 0 h control, one-way ANOVA with Tukey’s post-hoc test.

Since Nogo-A antibodies increase neurite outgrowth (Figure 3), we determined whether the antibodies would affect the expression of neurite outgrowth-promoting molecules. To this end, expression of the cell adhesion molecule L1 and polysialic acid were measured by western blot analysis at 2, 12, and 24 h after application of 5 and 1.25 μg/mL polyclonal Nogo-A antibody respectively. L1 and polysialic acid expression showed a tendency to increase at 1.25 μg/mL antibody concentration (Supplementary Figure S1A,B), being more prominent at 5 μg/mL (Figure 5 and Figure 6). At this concentration L1 expression was not increased by antibody application at 2 h, and tended to increase at 12 h to reach a statistically significant increase at 24 h (Figure 5A,B). Western blot analysis also showed that expression of polysialic acid increased in cell cultures treated with 5 μg/mL polyclonal antibodies at 12 and 24 h after application (Figure 6A,B). Untreated controls showed no difference in L1 or polysialic acid expression between 0 (Figure 5 and Figure 6) and 24 h in culture (Supplementary Figure S1A,B-lanes 1). 

Monoclonal Nogo-A antibody did not upregulate the expression of L1 and polysialic acid at both 1.25 and 5 μg/mL concentrations (Supplementary Figure S2A,B). These results show that polyclonal Nogo-A antibody enhances neurite outgrowth by regulating the expression of L1 and polysialic acid, whereas the effect of monoclonal Nogo-A antibody on neurite outgrowth is independent of regulation by L1 and polysialic acid expression. 

### 2.6. Nogo-A Antibodies Trigger Cell Signaling Pathways Required for Neurite Outgrowth 

**Figure 7:**
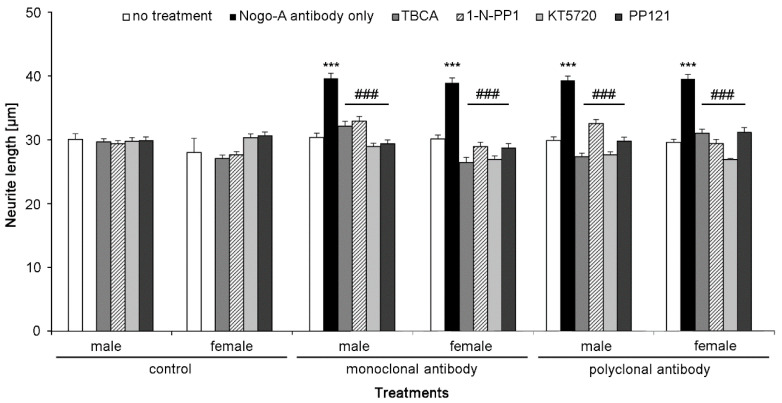
Nogo-A antibodies trigger cell signaling pathways underlying for neurite outgrowth. Bar diagram displays the average longest neurite length (mean + SEM; 300 neurons were analyzed for each treatment in three independent experiments) of male and female mouse cerebellar granule neurons pre-treated with different inhibitors of signal transducing molecules (TBCA (CKII), 1-N-PP1 (Fyn), KT 5720 (PKA), PP121 (Src)) or vehicle control-treated cells in the presence of 1.25 μg/mL monoclonal or polyclonal Nogo-A antibodies. Data represent mean  +  SEM. *** *p* < 0.001 difference to no treatment control, ### *p* < 0.001 difference of each treatment to Nogo-A antibody only control, one-way ANOVA with Tukey’s post-hoc test.

Since neurite outgrowth also depends on signal transducing molecules, we analyzed those known to underlie neurite outgrowth elicited by monoclonal and polyclonal Nogo-A antibodies. To this end, cultured cells were treated with inhibitors of different signal transducing molecules in the presence of Nogo-A antibodies. Neurite outgrowth observed with monoclonal and polyclonal Nogo-A antibodies was decreased with inhibitors of CKII, c-fyn, PKA, and c-src in both male and female cells (Figure 7). These results indicate that monoclonal and polyclonal Nogo-A antibodies trigger neurite outgrowth similarly via the signaling pathways examined in this study. 

## 3. Discussion

During the last decades, the functions of Nogo-A have received considerable attention in basic and translational neuroscience, since antibodies against it have been reported to promote recovery from different types of traumatic injury of the mammalian central nervous system [2,3,4,9,14,15]. A humanized monoclonal antibody is now in clinical trials. Nogo-A was originally reported to be a very prominent inhibitor of neurite outgrowth, being expressed at the oligodendrocyte cell surface, in myelin [2], and also in several populations of neurons [16]. The evidence that Nogo-A is not expressed in the mammalian peripheral nervous system was in agreement with the notion that regeneration after injury can occur, but when overexpressed in Schwann cells, it prevents regeneration after injury [17,18]. In the present study we investigated whether Nogo-A antibodies would be beneficial to neurons and therefore applied monoclonal and polyclonal antibodies to the prototypic culture of cerebellar granule neurons. Polyclonal antibodies are known to recognize multiple epitopes whereas a monoclonal antibody usually targets only one epitope. If the monoclonal antibody reacts specifically with the functionally important active site of a molecule, it is more efficient than the polyclonal antibody on a molar basis. It is important to point out that the epitopes of the monoclonal and polyclonal antibodies are in the N-terminal region of Nogo-A. The N-terminus is localized extracellularly when Nogo-A resides in the plasma membrane [19]. Nogo-A antibody staining of live cells assures that the antibodies interact with this molecule at the cell surface (see, for instance, [20]). Since gender differences have become important in present-day investigations, we have paid attention to this sensitivity. We show here that both types of antibodies affect neurons from male and female wild-type mice similarly. We deduce from our findings that male and female cells are not different in their response to Nogo-A antibodies. 

Several mouse mutations deleted in Nogo-A singly or together with the structurally-related Nogo-B and -C isoforms were reported to allow regeneration after injury, as shown by locomotor analysis and by inspection of the corticospinal tract [4,7,9]. However, it was also reported that mice ablated in these molecules, either singly or in combination with other myelin inhibitors, do not show enhanced recovery after spinal cord injury [10,21]. Furthermore, neurite outgrowth from NgR-deficient postnatal dorsal root ganglion or cerebellar granule neurons is inhibited by myelin and by a Nogo-66 substrate to the same extent as wild-type neurons [10]. These observations are reminiscent of the findings that mice deficient in the myelin-associated glycoprotein (MAG) did not show better axonal regrowth/sprouting/saving than their wild-type littermates, although MAG is an inhibitor of neurite outgrowth in vitro [22,23].

With the aim to investigate whether antibodies against Nogo-A would not only neutralize glial Nogo-A, but also affect neurite outgrowth and neuronal survival in vitro, we found that monoclonal and polyclonal antibodies against Nogo-A enhance neurite outgrowth as assayed in vitro with the paradigmatic mouse cerebellar granule neurons. These cultures are highly enriched in neurons and do not contain oligodendrocytes and astrocytes, when maintained for only two to three days in the presence or absence of serum [24]. Thus, if antibodies against Nogo-A enhance neurite outgrowth and neuronal survival, they can be considered to be function-triggering. The underlying cell signaling mechanisms essential for cell survival and neurite outgrowth are elicited by different signaling mechanisms [25,26]. In line with our findings, previous studies have shown that application of antibodies against Nogo-A to cultured dorsal root ganglion neurons enhance neurite outgrowth. These findings were verified in Nogo-A knock-out mice, where genetic ablation of Nogo-A throughout development increased neurite outgrowth [27]. Another study has shown that Nogo-A deletion did not reduce corticospinal axon regrowth/sprouting/saving after injury, whereas the sprouting capacity of oligodendrocytes was reduced [28]. These results supported the view that Nogo-A regulates neuronal regrowth. However, in a complex tissue it is difficult to attribute the observed effects to only a neuron-intrinsic capacity, particularly if Nogo-A was ablated throughout neuronal development, when compensatory mechanisms can occur. The signaling pathways underlying these effects were not investigated in these studies. In the present study we have analyzed signal-transducing mechanisms triggered by Nogo-A antibody and measured antibody-triggered expression of adhesion molecules that are known to enhance neurite outgrowth and neuronal survival. 

The bell-shaped dose–response curve seen in the present study is noteworthy and requires explanation. Such a response curve was observed with compounds mimicking the glycans LewisX and polysialic acid, which enhance neurite outgrowth at low concentration, but this effect was attenuated at higher concentrations [25,29]. Similarly, fibroblast growth factor 2 stimulates neurite outgrowth, and triggers concentration-dependent opposite responses in cultured cells with differential signaling, including negative feedback inhibition, that cause bell-shaped dose–response curves [30]. In this present study, the polyclonal Nogo-A antibody shows a bell-shaped outgrowth response, but was not toxic at higher concentrations, which indicates that a bell-shaped curve cannot be explained only by toxicity. 

The capacity of the antibodies to trigger beneficial neuronal functions was seen not only by their ability to enhance neurite outgrowth and neuronal survival, but also by their capacity to increase expression of the regeneration-conducive L1 cell adhesion molecule and polysialic acid glycan, which promote neurite outgrowth in vitro, synaptic plasticity, and regeneration after injury [31]. Furthermore, neuritogenesis fostered in the presence of Nogo-A antibodies could be inhibited by inhibitors of several signal-transducing molecules, thereby allowing a mechanistic interpretation of why these antibodies were acting agonistically together with several intracellular signal transducers that are known to contribute to neuronal survival and neurite outgrowth. 

Our results with rodent Nogo-A are in agreement with observations on regeneration-competent zebrafish which upregulate Nogo-A homolog expression in retinal ganglion cells after an optic nerve lesion, an effect that could be inhibited in vivo by antisense oligonucleotides targeting Nogo-A [32]. In addition to the intrinsic capability of retinal ganglion cells to regrow severed axons, zebrafish oligodendrocytes, and myelin-expressing Nogo-A lack the inhibitory functions of rodent myelin when offered, for instance, as a substrate barrier to neurites [32,33]. We conclude that intrinsic neuronal expression of Nogo-A is an important ingredient in regeneration after injury and expect that small compounds triggering the beneficial functions of neuronally-expressed Nogo-A will contribute to the amelioration of the severe consequences of central nervous system injury.

## 4. Materials and Methods 

### 4.1. Animals

CB6F1/J mice (Jackson Laboratory, Bar Harbor, ME, USA) were maintained for breeding with ad libitum access to food and water on a 12 h light and 12 h dark cycle in the pathogen-free animal facility of the Division of Life Sciences at the Nelson Biology Laboratories of Rutgers University. Six- to eight-day-old offspring of both sexes were used for cerebellar granule neuron culture. The Institutional Animal Care and Use Committee of Rutgers University approved all animal experiments (protocol no. 09-051, 10-May-2017).

### 4.2. Antibodies and Reagents

Chemicals were purchased from Sigma-Aldrich (St. Louis, MO, USA) if not indicated otherwise. Media and reagents for cell culture were purchased from Gibco (Gaithersburg, MD, USA). The casein kinase (CKII) inhibitor (E)-3-(2,3,4,5-tetrabromophenyl) acrylic acid (TBCA; Cat#sc-203869; CAS 934358-00-6) was from Santa Cruz Biotechnology (Dallas, TX, USA). c-src and c-fyn inhibitor 1-(1,1-dimethylethyl)-3-(1-naphthalenyl)-1H-pyrazolo [3-4-d] pyrimidin-4-amine (1-naphthyl PP1; Cat#529605; CAS 221243-82-9) and protein kinase A (PKA) inhibitor (9R, 10S, 12S)-2,3,9,10,11,12-hexahydro-10-hydroxy-9-methyl-1-oxo-9,12-epoxy-1H-diindolo [1,2,3-fg:3′,2′,1′-kl]pyrrolo[3,4-i][1,6]benzo-diazocine-10-carboxylic acid hexyl ester (KT 5720; Cat#1288/100U; CAS 108068-98-0) were from Tocris Bioscience (Bristol, UK). Calcein-AM (Cat#C1430; CAS 148504-34-1), and propidium iodide (Cat#P1304MP; CAS 25535-16-4) were from Thermo Fisher Scientific (Waltham MA, USA). Monoclonal mouse antibody to polysialic acid (PSA NCAM-1 antibody; Cat# NBP2-52710; lot#1521A03) was from Novus Biologicals (Centennial, CO, USA) and mouse antibody 172-R recognizing the intracellular L1 domain (anti-CD171; clone 74-5H7; Cat#38101) was from Biolegend (San Diego, CA, USA). Mouse glyceraldehyde 3-phosphate dehydrogenase (GAPDH) antibody (Cat#60004-1-Ig) was from Proteintech (Rosemont, IL, USA). The Nogo-A-specific mouse IgM monoclonal antibody (Nogo c-4; Cat#sc-271878; Lot#G0218; epitope mapping between amino acids 18-41 at the N-terminus of Nogo of human origin) and rat polyclonal antibody (Nogo-A; Cat#AF3098; Lot#WQH0119121; epitope mapping between amino acids Glu2-Val172) were, respectively, from Santa Cruz Biotechnology (Dallas, TX, USA) and R&D Systems (Minneapolis, MN, USA). Secondary anti-mouse IgG antibody coupled to horseradish peroxidase (HRP; Cat#715-035-150), secondary anti-mouse IgM antibody coupled to Alexa Fluor 488 (AF488; Cat#115-545-075), and secondary anti-rat IgG antibody coupled to AF488 (Cat#712-545-153) were from Jackson ImmunoResearch (West Grove, PA, USA).

### 4.3. Preparation of Dissociated Cerebellar Granule Neurons

Cerebellar granule neurons have a characteristic morphology of a small cell body, a minority of dendrites and a long process, which represents the axon. Purkinje cells are destroyed during cell preparation and do not survive, even after only 2 to 3 days of cell culture. Cerebellar granule neurons were prepared from 6- to 8-day-old mice as described by us [33]. In brief, the cerebellar cortices were cut into small pieces, which were then dissociated with trypsin and DNase for 15 min, washed with Hank’s balanced salt solution (HBSS), and then centrifuged at 100× *g* for 15 min at 4 °C. The pelleted cells were then cultured in serum-free Neurobasal-A medium (ThermoFisher Scientific, Waltham, MA, USA; Cat#10888022) supplemented with (Pen/Strep, 0.1% bovine serum albumin (BSA), 10 μg/mL insulin, 4 nM L-thyroxine, 100 μg/mL transferrin holo, 30 nM Na-selenite, Na-pyruvate, l-glutamine, and B-27) and maintained at 37 °C with 5% CO_2_ and 90% humidity. Cells from older mice cannot be prepared, because they are then more firmly integrated into the tissue, thereby destroying the cells during enzymatic and mechanical dissociation. Cells from younger mice were not prepared, because they yield insufficient numbers of cells per animal for biochemical analysis.

### 4.4. Immunostaining

Cerebellar granule cells were seeded onto glass coverslips coated with 0.01% poly-L-Lysine (PLL). After 24 h, the cells were stained live or after fixation [26]. For live cell staining, the cells were incubated with monoclonal or polyclonal Nogo-A antibodies (both at a 1:100 dilution of the stock solution) for 15 min at 37 °C. After three gentle washes with culture medium, the cells were fixed with 4% formaldehyde for 15 min at room temperature and blocked with 5% BSA dissolved in phosphate buffered saline, pH 7.3 (PBS) at room temperature for 1 h. We also fixed cells before primary antibody incubation. In this procedure, cells were first fixed with 4% formaldehyde for 15 min in culture medium at room temperature and blocked with 5% BSA dissolved in PBS at room temperature for 1 h. Cells were then incubated with monoclonal or polyclonal Nogo-A (both diluted 1:100) antibodies at 4 °C overnight. After 3 gentle washings in PBS, cells were stained with the corresponding secondary antibody coupled with Alexa Fluor 488. Thereafter, the coverslips were mounted with Fluoromount-G, with 4′, 6-diamidino-2-phenylindole (DAPI) (Invitrogen, Waltham, MA, USA; Cat# 00-4959-52). No detergent was used for this immunostaining procedure. Images were captured using a confocal microscope (LSM800, Carl Zeiss, Oberkochen, Germany). As background control, cells were treated only with the corresponding secondary antibodies.

### 4.5. Cell Survival

Cerebellar granule cells were seeded at a density of 1×10^6^ cells/mL (250 μL each well) in 0.01% PLL-coated 48-well flat-bottom tissue culture plates (Corning costar, Corning, NY, USA; Cat# 3548). To test which antibody concentrations are not toxic, cerebellar granule cells were treated with 1.25, 2.5, 5, 10, and 20 μg/mL monoclonal or polyclonal Nogo-A antibodies or vehicle control. We would like to mention here that the monoclonal antibody stock solution contains sodium azide. At an antibody concentration of 20 μg/mL, there was 0.3 mM sodium azide in the culture medium. The question of sodium azide functions has been studied in cultured proliferating cells [34]. Toxicity and mutagenesis were found at a concentration of 0.5 mM, when cells were treated with the agent for 16 h. Thus, for the present and previous studies, we can say that at the concentrations of sodium azide in our working solutions there is no toxic effect or mutagenesis.

To test if the Nogo-A antibodies increase cell survival under stress, cerebellar granule cells were maintained overnight at 37 °C with 5% CO_2_ and 90% humidity and subsequently treated with 1.25 μg/mL, 2.5 μg/mL, and 5 μg/mL monoclonal or polyclonal Nogo-A antibodies or vehicle control for 3 h. Oxidative stress was then induced by the addition of 10 μM H_2_O_2_ for 24 h.

Live and apoptotic cells were determined by incubation with 1 μg/mL calcein-AM and 1 μg/mL propidium iodide for 20 min at 37 °C. Live imaging of cells was performed using a Zeiss Axiovert 200M inverted transmission-light microscope (Carl Zeiss) with a × 20 objective and AxioVision 4.6 software (Carl Zeiss Microscopy LLC, White Plains, NY, USA). Numbers of living and apoptotic cells were determined for each image and the percentage of living cells was calculated. Experiments were carried out three times independently.

### 4.6. Neurite Outgrowth

Cerebellar granule cells were seeded (100,000 cells/mL; 100 μL/well) onto 0.01% PLL-coated 96-well Falcon tissue culture plates (Corning costar, Corning, NY, USA; Cat#353072) and treated with different concentrations of monoclonal and polyclonal Nogo-A antibodies (1.25 μg/mL, 2.5 μg/mL, and 5 μg/mL) or vehicle control. Cultures were also pre-treated for 20 min with different inhibitors of signal transducer molecules (120 nM KT5720, PKA inhibitor; 220 nM TBCA, casein kinase II inhibitor; 40 nM PP121, c-src inhibitor; 1.2 μM 1-naphthyl PP1, and c-fyn inhibitor) before Nogo-A antibodies (antibodies at 1.25 μg/mL) were added for 24 h at 37 °C in 5% CO_2_ and 90% humidity. Cells were then fixed at room temperature with 2.5% glutaraldehyde for 30 min and stained with 1% toluidine blue and 0.1% methylene blue in 1% Na-tetraborate. Neurites were imaged and quantified using an Axio Observer A1 microscope (Carl Zeiss Microscopy LLC, White Plains, NY, USA) with a × 20 objective and AxioVision 4.6 software. The longest neurite lengths were measured from the edge of the cell body to the end of the process, taking into account only neurites with a length equal to or greater than the diameter of the cell soma from which they originated and only from those that showed no contact with other neurites or cell bodies. To avoid the neurites from touching the neighboring cells, the cells were incubated with monoclonal and polyclonal Nogo-A antibodies for only 24 h. After longer culture times, neurites would be too long and thereby touch neighboring structures, preventing the accurate measurement of neurite lengths. Measurements were taken from 50 cells in each of two wells per condition analyzed using ImageJ software. Experiments were carried out three times independently.

### 4.7. Western Blotting

Cerebellar granule cells pooled from males and females were seeded into PLL-coated 6-well plates (VWR, Bridgeport, NJ, USA; Cat#10062-892) for 24 h and treated with 5 μg/mL monoclonal or polyclonal Nogo-A antibody for 2 h, 12 h, or 24 h or vehicle control. After two gentle washes in culture medium, cells were homogenized in RIPA buffer (20 mM Tris/HCl pH 7.4, 140 mM NaCl, 1% NP-40, 1 mM ethylenediaminetetraacetic acid (EDTA) and protease inhibitor cocktail, (Roche Diagnostics) and centrifuged at 20,000 ×  *g* for 10 min at 4 °C. Protein concentrations in the supernatants were determined with a bicinchoninic acid (BCA) assay (ThermoFisher Scientific, Waltham, MA, USA; Cat# 23225) and probes were mixed with sodium dodecyl sulfate (SDS) sample buffer (60 mM Tris/HCl, pH 6.8, 2% SDS, 1% β-mercaptoethanol, 10% glycerol, and 0.02% bromophenol blue) and incubated at 95 °C for 5 min. Equal quantities of proteins were separated by electrophoresis on a 4–12% Bis-Tris gel (Invitrogen, Waltham, MA, USA; Cat#NP0335BOX) and transferred to nitrocellulose membranes using iBlot 2 (ThermoFisher Scientific, Waltham, MA, USA; Cat#IB21001). Blots were incubated in a blocking solution of 5% BSA in 0.2% TBST (0.2% Tween-20 in Tris-base 0.1 M, pH 7.4) for 1 h at room temperature, and then incubated with primary antibodies. The membranes were washed and then incubated with L1 antibody 172 (diluted 1:1000), polysialic acid antibody 735 (diluted 1:1000) or GAPDH antibody (diluted 1:10,000) overnight at 4 °C. Secondary antibodies coupled to horseradish peroxidase were used at a dilution of 1:10,000. Immunoreactive bands were visualized using the advanced chemiluminescent substrate (GE Healthcare) and a gel imaging system LI-COR Biotechnology (Lincoln, NE, USA). The blots were quantified using ImageJ software. Each experiment was carried out three times independently.

### 4.8. Statistical Analysis

All experiments were performed and analyzed in a blinded manner. They were carried out three times independently. Average values and standard error of the mean (SEM) were calculated from a pool of at least three independent experiments. Statistical comparisons between groups were performed by one-way analysis of variance (ANOVA) using Fisher’s protected least significant difference (PLSD) test. StatView Version 5.0.1 (SAS Institute Inc., New York, NY, USA) and Microsoft Excel were used for all calculations.

## 5. Conclusions

Nogo-A and monoclonal antibodies against it have been intensely studied over many years in different lesion paradigms and were shown to ameliorate the severe outcome of central nervous system injury in adult mammals by neutralizing the inhibitory effects of Nogo-A expressed by oligodendrocytes and myelin. In the present study, we show that Nogo-A antibodies have an additional function, in that they directly enhance recovery-supporting functions in neurons, such as neurite outgrowth and neuronal survival. These supportive functions were exemplified by increased expression of the recovery-supportive cell adhesion molecule L1 and the glycan polysialic acid, both of which had been shown to ameliorate the consequences of traumatic injury in different lesion paradigms. Altogether these results allow a novel view on the functions of antibodies against Nogo-A.

## Supplementary Materials

Supplementary materials can be found at https://www.mdpi.com/1422-0067/21/15/5417/s1. Figure S1. Polyclonal Nogo-A antibody increases L1 and polysialic acid levels in cultures of cerebellar granule neurons. Cultures were maintained for 24 h under physiological conditions. Subsequently, neurons were treated with different concentrations (1.25 and 2.5 μg/mL) of polyclonal Nogo-A antibody and for different treatment times (2, 6, 12, and 24 h), or as control cultures not treated and maintained in culture for 24 h. Samples were collected after 24 h (lane 1) and evaluated for expression of (A) L1 and (B) polysialic acid (PSA) by western blot analysis using 172-R and 735 antibodies, respectively. As loading control, western blots were subsequently stained for GAPDH. 1.25 μg/mL (lanes 2–4): 6 h (lane 2), 12 h (lane 3), and 24 h (lane 4). 5 μg/mL (lanes 5–8): 2 h (lane 5), 6 h (lane 6), 12 h (lane 7), and 24 h (lane 8). Figure S2. Monoclonal Nogo-A antibody does not upregulate L1 and polysialic acid levels in cultures of cerebellar granule neurons. Cultures maintained for 24 h under physiological conditions. Subsequently, neurons were treated with different concentrations (1.25 and 2.5 μg/mL) of monoclonal Nogo-A antibody and for different treatment times (2, 6, 12, and 24 h), or as control neurons were not treated and kept in culture for 24 h. Samples were collected after 0 h (lane 1) and evaluated for (A) L1 and (B) polysialic acid (PSA) expression by western blot analysis using 172-R and 735 antibodies, respectively. As loading control, western blots were subsequently stained for GAPDH. 1.25 μg/mL (lanes 2–4): 6 h (lane 2), 12 h (lane 3), and 24 h (lane 4). 5 μg/mL (lanes 5–8): 2 h (lane 5), 6 h (lane 6), 12 h (lane 7), and 24 h (lane 8).

## Abbreviations

ANOVA	analysis of variance
BCA	bicinchoninic acid
BSA	bovine serum albumin
CKII	casein kinase II
DAPI	4′,6-diamidino-2-phenylindole
DRG	dorsal root ganglion
EDTA	ethylenediaminetetraacetic acid
GAPDH	glyceraldehyde 3-phosphate dehydrogenase
H_2_O_2_	hydrogen peroxide
HBSS	Hanks balanced salt solution
PBS	phosphate buffered saline
PLL	poly-L-lysine
PLSD	protected least significant difference
SDS	sodium dodecyl sulfate
SEM	standard error of the mean
TBST	Tween-20 in Tris-base
