# Supplementary material for: Application of Antibodies to Neuronally Expressed Nogo-A Increases Neuronal Survival and Neurite Outgrowth
Source: Int J Mol Sci. 2020 Jul 30;21(15):5417. doi: 10.3390/ijms21155417 (PMC7432704; doi:10.3390/ijms21155417)
Supplement: Supplementary file 1 [file ijms-21-05417-s001.pdf]

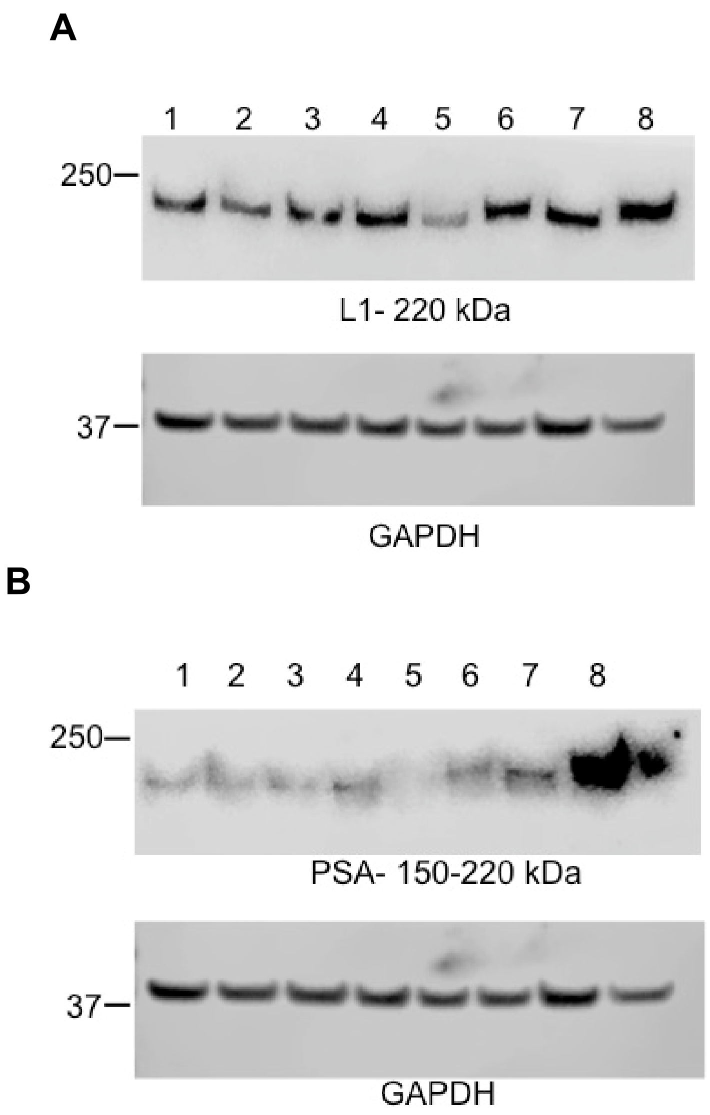

Supplementary Figure 1. Polyclonal Nogo-A antibody increases L1 and polysialic acid levels in cultures of cerebellar granule neurons. Cultures were maintained for 24 hour under physiological conditions. Subsequently, neurons were treated with different concentrations (1.25 and 2.5 µg/ml) of polyclonal Nogo-A antibody and for different treatment times (2, 6, 12, and 24 hour), or as control cultures not treated and maintained in culture for 24 hour. Samples were collected after 24 hour (lane 1) and evaluated for expression of L1 (A) and polysialic acid (PSA) by Western blot analysis using 172-R and 735 antibodies, respectively. As loading control, Western blots were subsequently stained for GAPDH.

1.25 µg/ml (lanes 2-4): 6 hour (lane 2), 12 hour (lane 3) and 24 hour (lane 4)

5 µg/ml (lanes 5-8): 2 hour (lane 5), 6 hour (lane 6), 12 hour (lane 7) and 24 hour (lane 8)

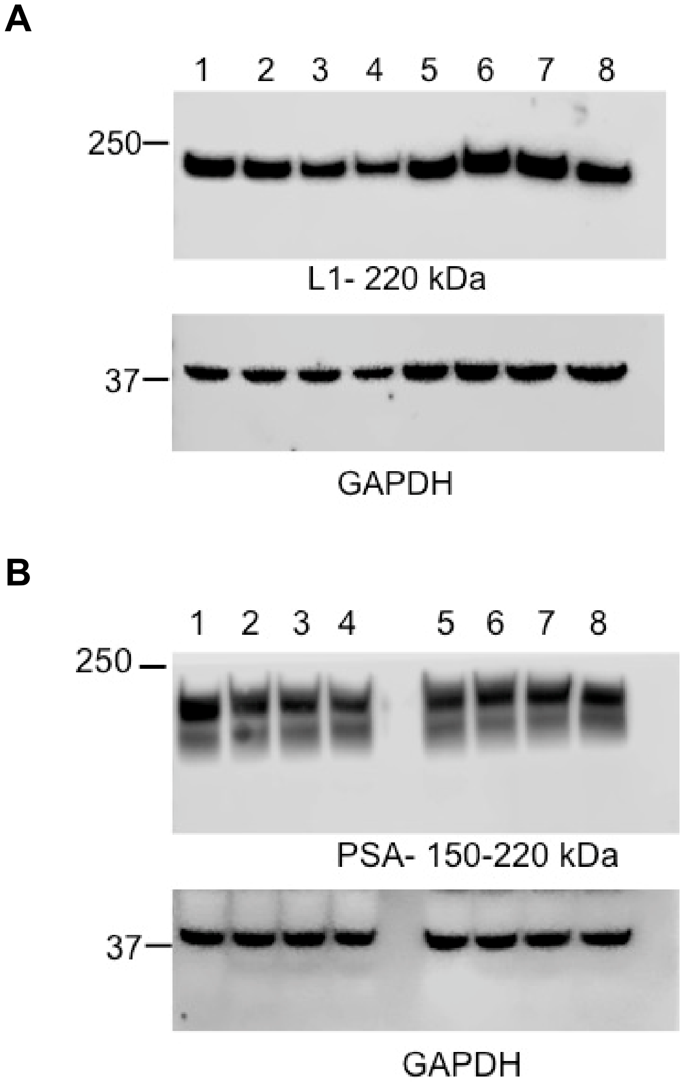

Supplementary Figure 2. Monoclonal Nogo-A antibody does not upregulate L1 and polysialic acid levels in cultures of cerebellar granule neurons. Cultures maintained for 24 hour under physiological conditions. Subsequently, neurons were treated with different concentrations (1.25 and 2.5 µg/ml) of monoclonal Nogo-A antibody and for different treatment times (2, 6, 12, and 24 hour), or as control neurons were not treated and kept in culture for 24 hour. Samples were collected after 0 hour (lane 1) and evaluated for (A) L1 and (B) polysialic acid (PSA) expression by Western blot analysis using 172-R and 735 antibodies, respectively. As loading control, Western blots were subsequently stained for GAPDH.

1.25 µg/ml (lanes 2-4): 6 hour (lane 2), 12 hour (lane 3) and 24 hour (lane 4)

5 µg/ml (lanes 5-8): 2 hour (lane 5), 6 hour (lane 6), 12 hour (lane 7) and 24 hour (lane 8)
